# Insights on seed abortion (endosperm and embryo development failure) from the transcriptome analysis of the wild type plant species Paeonia lutea

**DOI:** 10.6026/97320630016638

**Published:** 2020-08-31

**Authors:** Shanshan Zhang, Yuanhui Li, Yuanrong Li, Fan Zhao, Xiuli Zeng

**Affiliations:** 1 The Ministry of Agriculture of Qinhai-Tibet plateau Fruit Trees Scientific Observation Test Station, Lhasa, Tibet 850032, China; 2 Institute of Vegetables, Tibet Academy of Agricultural and Animal Husbandry Sciences, Lhasa, Tibet 850002, China

**Keywords:** Paeonia lutea, Seed abortion, Transciptome

## Abstract

Paeonia lutea is a wild peony (an endangered flowering plant species) found in China. Seed abortion (endosperm and embryo development failure) is linked to several endangered plant species. Therefore, it is of interest to complete a comparative analysis of transcriptome between the normal active seeds (Population A) and the endangered abortion seeds (Population H). Data from GO assignments of differentially expressed genes (DEGs) shows that "metabolic process", "binding", "cellular process", "catalytic activity", "cell" and "cell part" are commonly prevalent in these popuations. DEGs between the populations are found to be connected with metabolic pathways, biosynthesis of secondary metabolites, purine metabolism and ribosome. We used quantitative RT-PCR to validate 16 DEGs associated with these populations. It is found that histone genes and proline-rich extensin genes are predominant in the common groups. Histone genes (H2A, H2B, H3, H4 and linker histone H1) show 3 to 4 folds log2FC higher expession in population A than in population H in stage I unlike in stage II and III. Increased activity of proline-rich extensin genes in population A than in population H corresponding to seed abortion in the later population is implied. These preliminary data from the transcriptome analysis of the wild type plant species Paeonia lutea provide valuable insights on seed abortion.

## Background

Tree peony (Paeonia suffruticosa Andrews) is an important, traditional and most well-known ornamental and medicinal plant in the world due to its colorful flowers and medicinal values, which belongs to Moutan subfamily, the genus Paeonia, family Paeoniaceae [1,2]. Paeonia lutea with special bright yellow flowers and large plant size (1.1-2.3 m) obviously distinguished it from other species of tree peony. The flower colour of most tree peony species is pink, red, purple-red, or white [3,4], bright yellow is rare in the tree peony cultivars. Thus, Paeonia lutea is considered to be the most precious resource for tree peony cultivar breeding [1]. It was classified as rare and endangered plants in 1987 in China. It distributed in middle and northwestern of Yunnan province, southwestern of Sichuan province and Tibet. It usually grows in mountains with elevation of 2500-3500m, the distribution area is very narrow. It has been reported that seedling numbers and total plant numbers of Paeonia lutea in natural environments declined year by year during the past 20 years [5]. It is endangered for its small quantity and narrow distribution. Natural reproduction of Paeonia lutea is mainly by root suckers and seeds, and most of the populations can breed with seeds by themselves and keep a normal growth state. While some populations has been observed with seed abortion problem, the seeds of these plants were small, thin and showed extremely low activity. It is severe for its propagation and may exacerbate its endangering rate. However, there are very few researches focused on its seed abortion mechanisms.

Seed development was been regulated by both exogenous and endogenous factors. For the two kinds of Paeonia lutea population (normal populations with active seeds and seed abortion populations) in our test, since they are distributed in the same environment with similar climatic conditions, the exogenous factors might not be the major driving forces of seed abortion in Paeonia lutea. The endogenous factors as the key to seed formation are regulated by genes expression or repression during the development processes. However, there is no report on the genes or involved pathways on seed development of Paeonia lutea so far. Therefore, it is of interest to complete a comparative analysis of transcriptome between the normal active seeds and the endangered abortion seeds to derive meta-data for explaining seed abortion in Paeonia lutea.

## Methodology

### Plant materials:

**Figure 1:**
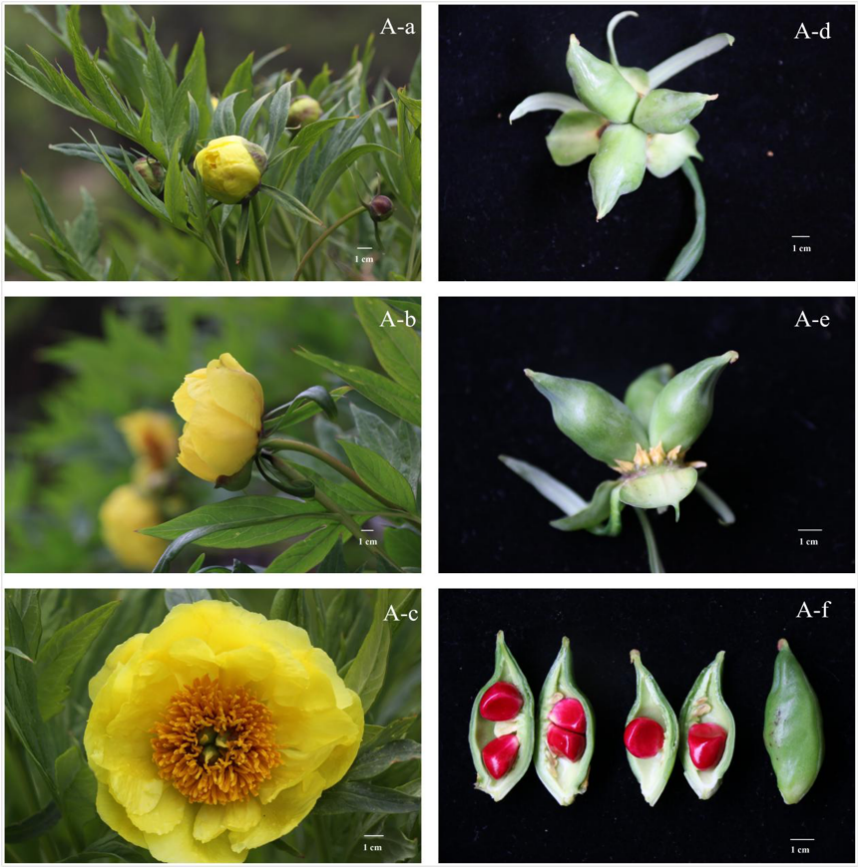
Normal seed formation of wild Paeonia lutea populations; Note: A-a, A-b and A-c showed three sampling time-point of stage I (flower bud three days before blooming), II (initial blooming time, before pollen dispersion) and III (eight days after pollination) in normal populations; A-d, A-e and A-f showed the active seeds in normal populations Latitude of population H: 94°32'69" E, 29°67'48" N. Date: A-a: May 6th, 2016. A-b: May 3th, 2016. A-c: May 14th, 2016. A-d, A-e, A-f: June 20th, 2016.

**Figure 2:**
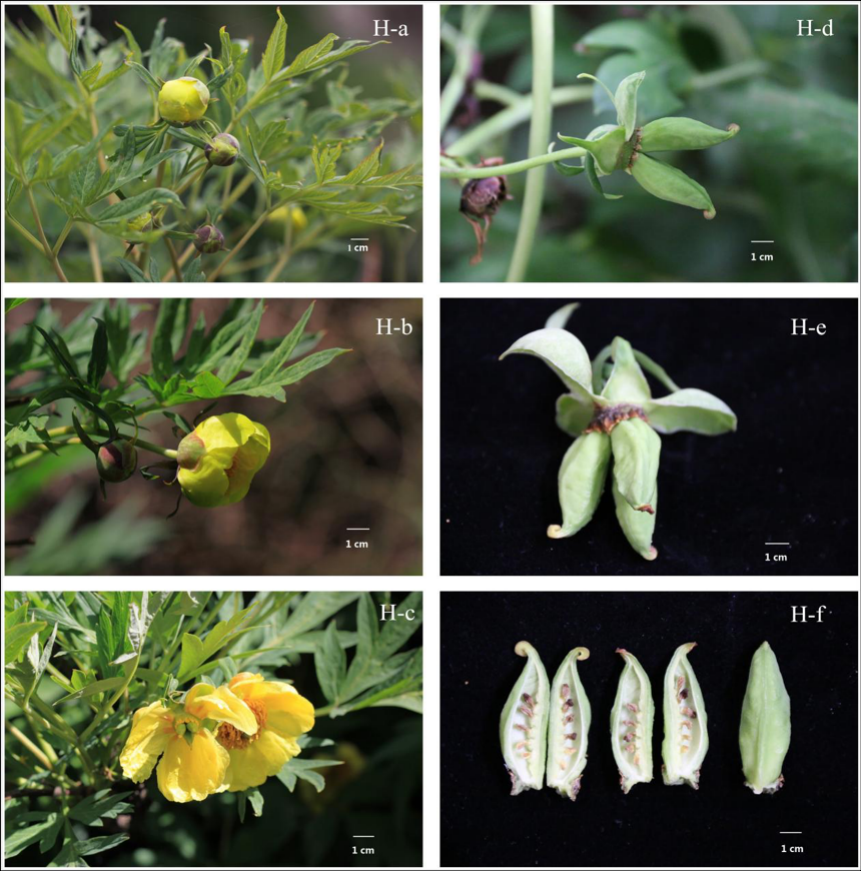
Seed abortion of wild Paeonia lutea populations Note: H-a, H-b and H-c showed three sampling time-point of stage I (flower bud three days before blooming), II (initial blooming time, before pollen dispersion) and III (eight days after pollination) in seed abortion populations; H-d, H-e and H-f showed the aborted seeds in population H. Latitude of population H: 94°47'74" E, 29°54'65"N. Date: H-a: May 6th, 2016. H-b: May 3th, 2016. H-c: May 14th, 2016. H-d, H-e, H-f: June 20th, 2016.

**Figure 3:**
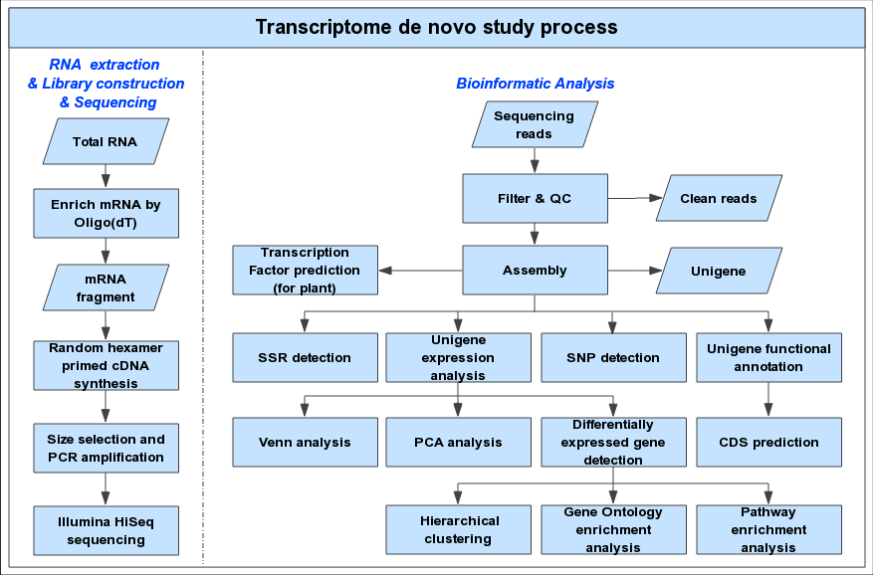
The workflow for sequencing and bioinformatic analysis

The experiments were conducted at Nyingchi Prefecture (29°34'N, 94°37'W), Tibet, China, using wild Paeonia lutea populations as plant materials. Two populations of wild Paeonia lutea with contrasting seed performance (normal vs. abortion, referred to as Population A and H, respectively) were used for artificial pollination. Ten individuals of each population were chosen randomly for pollination. Sampling method was as follows: Flower bud, blooming flower, and pollinated flower were sampled at three stages: stage I, flower bud three days before blooming (Figure 1A-a;Figure 2H-a), stage II, Initial blooming time, before pollen dispersion (Figure 1A-b;Figure 2H-b), same time to implement artificial pollination; stage III, eight days after pollination (Figure 1A-c;Figure 2H-c). At each stage, the two populations were sampled at the same time with three biological replicates. All samples were immediately frozen in liquid nitrogen and stored in -80°C refrigerator for RNA extraction. The workflow for sequencing and bioinformatic analysis are given (Figure 3).

### cDNA construction and sequencing:

Total RNA of 18 samples was extracted using the CTAB method, then removed contaminating genomic DNA with RNase-free DNase I (TIANGEN, TIANGEN BIOTECH (BEIJING), China) according to manufacturer's standard protocols. The RNA quality was controlled using Nanodrop, Qubit 2.0 and Aglient 2100. After that, RNA was used for cDNA library construction. The mRNA was enriched by magnetic beads with Oligo (dT), mixed with fragmentation buffer, and then fragmented into short fragments in Eppendorf ThermoMixer® C (Eppendorf, Germany). The short fragments were used to synthesize first-strand cDNA and double-strand cDNA. The double-strand cDNA was purified with Qia-Quick PCR extraction kit (Qiagen; Valencia, CA, USA). Quality control of libraries was determined with Agilent 2100 Bioanalyzer (Agilent Technologies; Palo Alto, CA, USA) and an ABI StepOnePlus Real-Time PCR System (Applied Biosystems; Foster City, CA, USA). The cDNA libraries were sequenced by a HiSeq 2500 sequencing platform (Illumina Inc.; San Diego, CA, USA) at Beijing Genomics Institute (BGI).

### De novo assembly of Paeonia lutea transcriptome:

Firstly, the raw reads were filtered by discarding adaptor sequences, low quality reads, reads with adaptors and reads in which unknown bases (N) are more than 5% were removed to get clean reads. Then, Clean reads will be assembled into unigenes using the Trinity software with an optimized k-mer length of 25 [6].

### The identification of differential expressed genes and pathway analysis:

Clean reads were mapped to unigenes using Bowtie2 (v2.2.5) [7], and then calculated gene expression level of unigenes with RSEM (v1.2.12) [3]. Differential expressed genes (DEGs) were detected with NOIseq [8] as requested, with parameters of Fold Change >= 2.00 and Probability >= 0.8. DEGs were aligned by Blastx to public databases for functional annotations. Databases including NCBI non-redundant protein database (NR), Swiss-Prot protein database and Orthologous Group (COG) were used for gene annotation. Gene ontology (GO) database was used to obtain the relevant GO terms of DEGs for functional classification. Kyoto Encyclopedia of Genes and Genomes (KEGG) database was used for pathway analysis. The False Discover Rate (FDR) <0.01 was the threshold for the hypothesis testing.

### Quantitative Real-Time PCR Analysis:

qRT-PCR was performed for 16 candidate DEGs for further validation. RNA extraction and purification of all samples were performed as described above. The first-stand cDNA was synthesized from 4µg of DNA-free RNA using reverse transcription system (Prime Script RT reagent Kit Perfect Real Time) (Takara, Japan). qRT-PCR reactions were performed using SYBR Premix Ex Taq (Takara, Japan) on ABI 7500 Real-Time system (Applied Biosystems, USA).The amplification program of qRT-PCR was performed in a volume of 20 µl containing 10 µl SYBR Premix Ex Taq, 2 µl cDNA, 1 µl of each primer, and 6 µl RNase-free sterile water. PCR reactions were performed at 95°C for 2min, followed by 40 cycles of 95°C for 15 s and 60°C for 31 s. All qRT-PCR reactions were performed with three biological replicates. The relative expression levels of all selected genes were calculated using the 2¯ΔΔCT method [9]. GAPDH was used as reference gene to normalize the relative expression of selected genes [10]. The qRT-PCR results were compared with the results of transcriptomic analysis. Primers used for candidate genes were listed in Table 1.

## Results

Paeonia lutea populations in this experiment were originated in Tibet, and this experiment were conducted in Nyingchi Prefecture (29°34'N, 94°37'W), Tibet, China. In this distribution area, some populations have been investigated regarding to seed abortion problems. According to the survey, in these populations, almost all seeds were aborted in each individual (data not shown). The seed coat of normal populations was plumpness while the aborted seeds were small, thin and flat (Figure 1A-d,e;Figure 2H-d,e). The ovules were aborted completely in the group H, while 2 to 4 ovules in each pod developed into active seeds successfully in group A (Figure 1A-f;Figure 2H-f).

## Transcriptome Sequencing and Assembly:

Sequencing projects generated 120.91 Gb raw data from 18 libraries by Illumina Hiseq 2500 instrument. After removal of adaptor sequences, ambiguous reads, low quality reads, and assemblling all samples together, 155,685 unigenes were acquired. The total length, average length, N50, and GC content of Unigenes were 140,639,935 bp, 903 bp, 1,525 bp, and 40.72 %, respectively (Table 2). This raw sequencing data is available at the NCBI Sequence Read Archive (SRA) database under accession of PRJNA545629.

## Unigenes functional annotation and classification:

**Figure 4:**
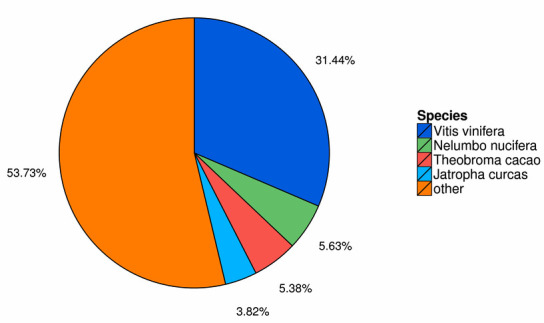
Species distribution of unigenes of Paeonia lutea

All assembled unigenes were aligned to seven public functional databases to identify the putative functions with an E-value cut off of 1e. In total, 79,140(50.83%) unigenes in the de novo transcriptome libraries showed significant similarity to known proteins. Unigenes annotation information in seven databases were shown in Table 3. For species distribution, 21621 (31.44%), 3875 (5.63%), 3703 (5.38%) and 2625 (3.82%) assembled transcripts were aligned to Vitis vinifera, Nelumbo nucifera, Theobroma cacao and Jatropha curcas, respectively (Figure 4). Additionally, due to the absence of Paeonia genome and gene sequences in public databases, only 322 unigenes were mapped to Paeonia, of which 128 and 121 unigenes sequences shared high similarity with genes of Paeonia lactiflora and Paeonia suffruticosa, respectively.

## Seed formation related DEGs selection and functions annotation in Paeonia lutea:

**Figure 5:**
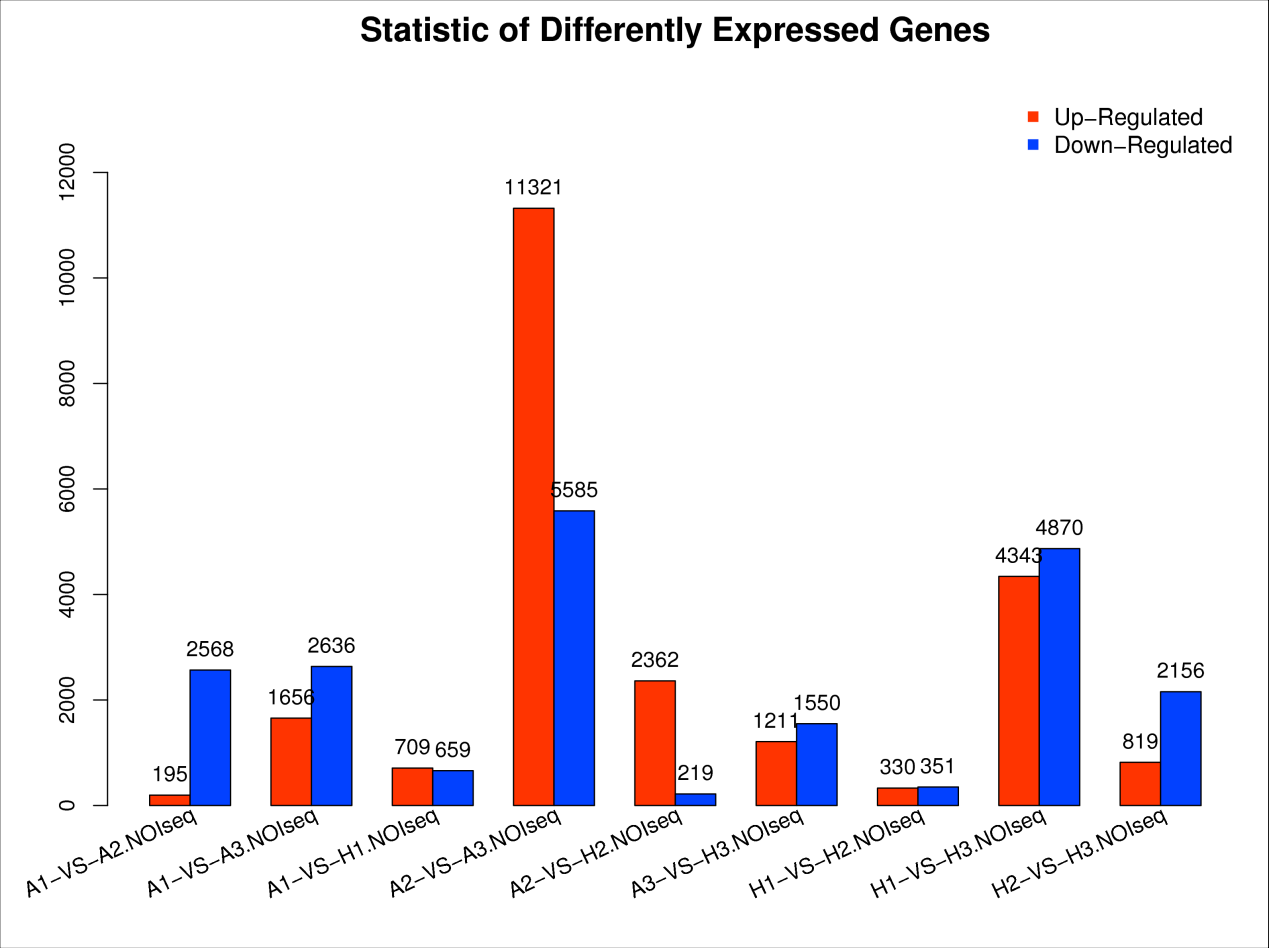
Statistic of Differently Expressed Genes

To identify the candidate genes controlling seed formation and differentially expressed between normal populations and seed abortion populations, we performed differentially expressed gene (DEG) analysis by NOIseq [8]. The parameters of False were used as threshlds Discovery Rate (FDR) ≤ 0.001 and Fold Change (log 2 ratio) ≥1 to select DEGs during the three stages of reproduction (A1 vs. H1, A2 vs. H2, and A3 vs. H3). Transcriptome profiling of A1 vs. H1 tissues obtained 1,368 DEGs, with 709 unigenes significantly up-regulated while 659 unigenes down-regulated. A total of 2,581 unigenes were identified differentially expressed between A2 and H2, with 2,362 up- regulated and 21 down-regulated unigenes. Total of 2,761 unigenes were identified as DEGs between A3 and H3, with 1,211 up-regulated and 1,550 down-regulated unigenes (Figure 5).

**Figure 6:**
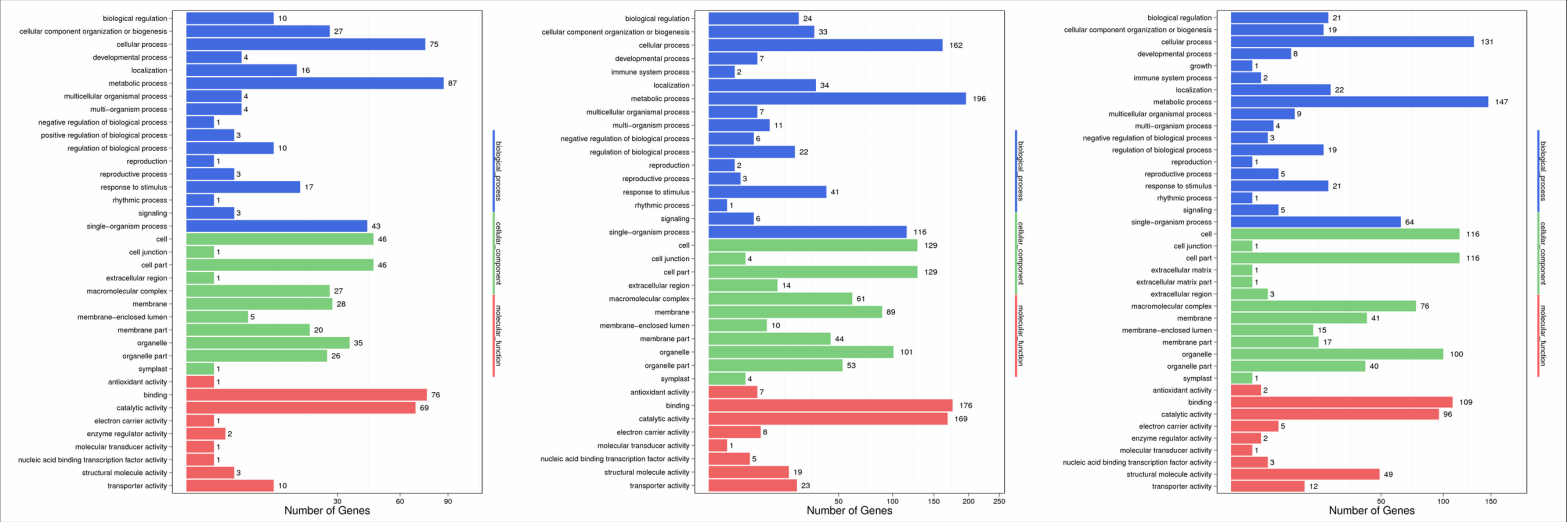
GO assignments of DEGs between population A and H at three stages

GO assignments were performed to clarify the functions of the DEGs in three comparison groups (A1 vs. H1, A2 vs. H2, and A3 vs. H3). In GO database, DEGs of A1 vs. H1, A2 vs. H2, and A3 vs. H3 were distributed into "cellular process", "catalytic activity" and "metabolic process" with total of 37, 36 and 40 annotation categories, respectively. In general, the DEGs distributed GO annotation categories were similar overall in three groups. The most common enriched items for the three groups were "metabolic process" (87, 196, and 147 DEGs distributed in A1 vs. H1, A2 vs. H2, and A3 vs. H3, respectively), "binding" (76, 176, and 109), "cellular process" (75, 162, and 131), "catalytic activity" (69, 169, and 96), "cell" (46, 129, and116), and "cell part" (46, 129, and 116) (Figure 6).

## KEGG pathway analysis of seed formation related DEGs in Paeonia lutea:

**Figure 7:**
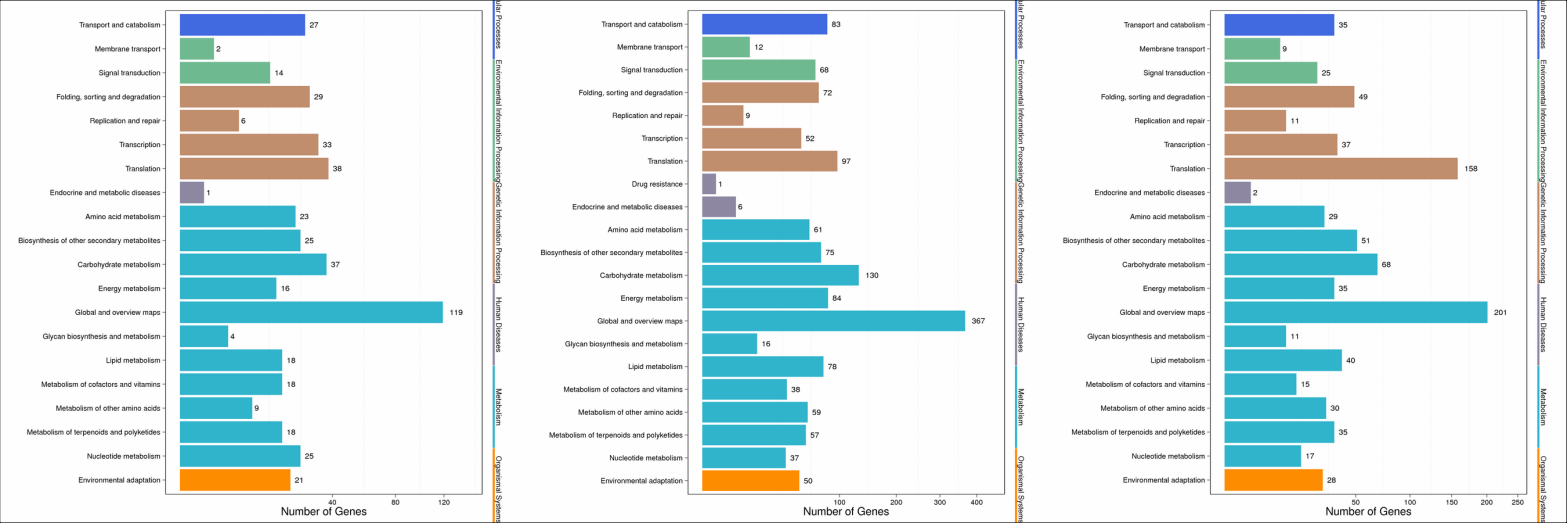
KEGG pathway analysis of DEGs between population A and H at three stages

At stage I, between A1 and H1, 242 unigenes were assigned to 6 main categories including 108 pathways. Stage II (H2 vs. A2) specific enriched pathways included "Sphingolipid metabolism" (7), "Propanoate metabolism" (5), "Brassinosteroid biosynthesis" (5), "Base excision repair" (4), "DNA replication" (4), "Mismatch repair" (3), "Glycosphingolipid biosynthesis-ganglio series" (3), "Glycosphingolipid biosynthesis - globo series" (2), "Glycosaminoglycan degradation" (2), "Fatty acid elongation" (2) and "Taurine and hypotaurine metabolism" (2) Stage III (H3 vs. A3) specific enriched pathways included "Anthocyanin biosynthesis" (10), "Synthesis and degradation of ketone bodies" (6), "Monoterpenoid biosynthesis" (6), "Monobactam biosynthesis" (3), "Thiamine metabolism" (3), "One carbon pool by folate" (3) and "Lysine biosynthesis" (2) (Figure 7). Overall, Stage II was the most active phase according to the pathway analysis.

There were 11 common enriched pathways with large number of DEGs in all three stages, which included "Metabolic pathways" (117, 347, and 183 DEGs in H1 vs. A1, H2 vs. A2, and H3 vs. A3, respectively, the same below), "Biosynthesis of secondary metabolites" (62, 197, and 109), "Purine metabolism" (23, 28, and 14), "Ribosome" (7, 52, and 109), "Pyrimidine metabolism" (23, 26, and 11), "Plant-pathogen interaction" (19, 45, and 24), "RNA transport" (17, 25, and 29), "Plant hormone signal transduction" (11, 44, and 22), "Glycolysis/ Gluconeogenesis" (10, 27, and 14), "Carbon metabolism" (9, 49, and 23), and "Biosynthesis of amino acids" (13, 30, and 19) (Figure 6).

## qRT-PCR validation of core candidate DEGs from RNA-Seq:

**Figure 8:**
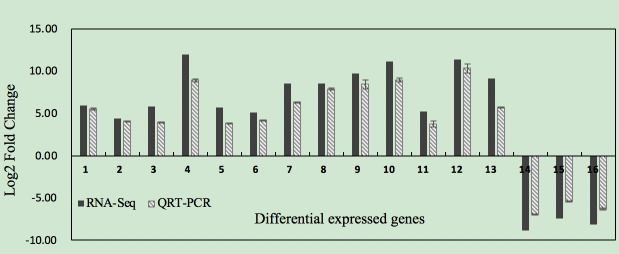
Gene expression of qRT-PCR and RNA-Seq results of 16 DEGs Note: DEGs ID: 1, Unigene9864; 2, Unigene32386; 3, Unigene28757; 4, Unigene28168; 5, Unigene10363; 6, CL9171; 7, CL9299; 8, Unigene39677; 9, CL2592; 10, CL1367; 11, CL12495;12, CL2588; 13, CL4787; 14, CL6183; 15, CL5372; 16, CL1009.

To confirm the accuracy and reproducibility of the Illumina RNA-Seq results, 16 core candidate DEGs were verified using qRT-PCR. The RNA-Seq results and qRT-PCR values were displayed in Figure 8, showing consistent expression patterns for those candidate DEGs.

## Discussion

Paeonia lutea as the most precious resource for tree peony cultivar breeding, it is endangered for small quantity and narrow distribution. Natural reproduction of Paeonia lutea in wild is mainly by seeds while some populations have been found with severe seed abortion problem. In this study, transcriptome comparative analysis between the sexual reproductive abortion population and the normal population of Paeonia lutea was carried out to explore the possible mechanism of seed abortion.

Paeonia lutea belongs to Moutan subfamily, the genus Paeonia, family Paeoniaceae. Compared with those model plants, its genomic research is limited, and the relative biological information is insufficient. It is the first time to study the genomics of Paeonia lutea. Therefore, de nove assembly technology was used to assemble the transcripts of Paeonia lute. The overall annotation rate of Unigenes was 50.83%, which is very low. Nearly half of the genes could not be annotated effectively. This indicates the unique genome information that the yellow peony may have. The large amount of gene expression information data obtained in this study will greatly enrich the genetic data resources of the yellow peony, which will provide a basis for the further study of the yellow peony on molecular level.

Seed abortion in natural plants has been noticed and discussed for a long time. Bawa et al.[11] pointed out that there are several hypotheses on seed abortion in natural populations of plants. Parent-offspring conflict over resource allocation, sibling rivalry, pollen competition and genetic load theory had been proposed. These theories explained seed abortion in some plants successfully with an exception in an endangered plant named polygonaceae (Dedeckera rurekensis), this plant had been observed with 97.5% percent of seed developmental failure [12], which was not randomly occurred among the seeds apparently, so it cannot be well explained by any of the above hypotheses. Similarly, the seed abortion phenomenon of Paeonia lutea in natural populations in Tibet is just like what happened to polygonaceae, almost 100% of seeds was aborted in some populations. Sun et al.[13] reported that environment stresses could be the key reasons that lead to seed abortion, however, the normal populations and the seed abortion populations of Paeonia lutea are in the same habitat, excluding the environmental factors. Thus, the inherent genetic reasons may be involved. Urgent study needs to find the reason in case the situation becoming more severe.

Hence, in this study, transcriptome comparative analysis was applied between the sexual reproductive abortion population and the normal population of Paeonia lutea, aimed to explore the related genes or pathways, which may explain the seed abortion problem. Three key stages during reproductive development process were chosen in this experiment, stage I, Flower bud three days before blooming; stage II, initial blooming time before pollen dispersion and stage III, eight days after pollination. Stage II was showed to be the most activity phase during the whole process through the transcriptome test.

The results suggested that histone genes may involve in the reproductive development processes in Paeonia lutea, a group of DEGs on histone proteins were notable in our test (Table 4). As it showed in the table, during stage I, there were 11 DEGs annotated as histone H2B, histone H2A, histone H3 and histone H1, and the expression level of all DEGs was 3.7-4.3 log2FC in group A than in group H. During stage II, there were 8 DEGs annotated as histone H3, histone H1 and histone H2B, while the gene expression level was opposite to stage I, it was 3.9-4.1 log2FC in group H than in group A. During stage III, there were 3 DEGs annotated as histone deacetylase HDT1, histone-binding protein RBBP4 and histone H1, and the expression level of genes in group H was significantly higher than that in group A. There seemed showing a pattern that histone proteins were produced earlier in normal seed formation plants than in seed abortion plants.

Histone proteins including core histones H2A, H2B, H3, H4 and linker histone H1, DNA was wrapped around an octamer of histone proteins to form nucleosomes, and the changes of histone proteins lead to higher order chromatin structure formation and remodeling [14,15,16]. Histone modifications including methylation, acetylation, phosphorylation, ubiquitination, and sumoylation, would alter nucleosome stability and positioning, and then affect DNA accessibility for regulatory proteins or protein complexes involved in transcription, DNA replication and repair [17,18, 19]. Studies have unraveled diverse epigenetic regulatory mechanisms involved in different processes during floral organogenesis and sexual reproduction in Arabidopsis and rice [1,20]. Histone H3 methyltransferase is required for ovule development in Arabidopsis [21]. It can be inferred that during the reproductive process, histones activity was highly correlated with the expression of key function genes on reproductive regulation. In our test, histone genes were induced highly in stage I in group A uniformly, while in Stage II and III they were highly induced in group H uniformly. The difference of histones proteins dynamic between group H and group A may lead to different seed formation process, while their exactly regulation role on seed development still need to be explored in the future study.

The plant proline-rich proteins, which belonged to a class of proline and hydroxyproline-rich proteins and mainly localized in the cell wall, have been pointed out to act on seed developmental program and coordinate the physiological events occurring during celluar process [22,23]. It expressed specifically in different tissues and developmental stages, and has been reported to regulate cell wall structure in plants [23] In this test, a group of proline-rich extension proteins were selected as DEGs (Table 5), they showed different patterns in group A and group H during floral organ development process. Generally, the genes’ expression level was much higher in group A than in group H, especially in stage III. Unigene9091and other 7DEGs which annotated as extensin-like protein were highly induced in group A. some researchers concluded that SbPRP1 was one of the highly expressed forms of cell wall proteins at the stage of seed coat development [24]. Four days after fertilization, over one hundred genes were identified with exclusively high expression in young seed stages, and most of these genes were annotated as histones and proline-rich proteins [25]. The proline-rich proteins may act as key regulating factors in seed cell development, for their activity in group A was much more intense than that in group H, which may cause seed cell development disorder in group H, then lead to seed abortion.

## Conclusion

We report the predominant presence, activity and expression of histone genes (H2A, H2B, H3, H4 and linker histone H1) and proline-rich extensin genes linking to seed abortion in Paeonia lutea using DEG data in stage I unlike in stage II and III. These data from the transcriptome analysis of the wild type plant species Paeonia lutea provide valuable insights on seed abortion towards improved crop management.

## Declaration on Publication Ethics:

The authors state that they adhere with COPE guidelines on publishing ethics as described elsewhere at https://publicationethics.org/. The authors also undertake that they are not associated with any other third party (governmental or non-governmental agencies) linking with any form of unethical issues connecting to this publication. The authors also declare that they are not withholding any information that is misleading to the publisher in regard to this article.

The authors are responsible for the content of this article. The Editorial and the publisher has taken reasonable steps to check the content of the article with reference to publishing ethics with adequate peer reviews deposited at PUBLONS.

## Figures and Tables

**Table 1:** Primers used in this test

Candidate genes		5'to3'
Unigene28757	F	GATCAAGCGTCTTGGTGACA
	R	TGCTGAAGAAAGCGTACGAA
Unigene9864	F	GGAAGCCACAACTTGGAGAA
	R	ACTTGACTACCCGCAGGAGA
Unigene32386	F	TCAGTCTTGTGTGATGCTGAG
	R	GACTCAATGAGTCAAGATCT
Unigene28168	F	GGCCTTTAGTTCGTTCCACA
	R	AGCTCTCGGGTTGGAGCTAT
Unigene10363	F	ATCCACACAATGGGGAAAAA
	R	ATGAATGCTGGGTTTTGAGG
CL9171	F	GTGTCGTGACGATGACCACTG
	R	ACCAAGTTGTTGGTCCATATG
CL9299	F	ACTCGCGACGCGCTCTCCAGG
	R	GCCTCGTTGACAGGAGGACA
Unigene39677	F	CAAGATTCTATCGGGCTTGG
	R	CGTGCCTCTATGTGGCTGTA
CL2592	F	TCACAACTTCAAGCATGGTTG
	R	GACTCAGAAGACTCCCTCGAT
CL1367	F	AGACTCAGCTACTGTCCAGG
	R	ACCCACCTGGCTATGTGATC
CL12495	F	AGATGGATTCTGATCGTCTG
	R	CATTAGCTAAAGCAGATGAAG
CL2588	F	AGTATTGAGACTAGTGGGTGC
	R	GTCAGCATGTTATTCTCAGCA
CL4787	F	ACGGCGATAGATATCGAGGAT
	R	TAGTCTTAGTGACAGCTGCTG
CL6183	F	ATGATGTTCACCAGGTATGG
	R	TGTGTAATCAACTAATCACG
CL5372	F	CAGGAGCTAGAGATGCTATC
	R	ACCTAGCATTGCGCTGGAGC
CL1009	F	ACACAGATGGCGATGCCGACG
	R	GAGATGCACTGAAGAAGCATG
PS-GAPDH	F	GGTTGATCTCACTGTTAGGC
	R	TCAGACTCCTCCCTACAAG

**Table 2:** Summary of transcriptome sequencing and assembly results of Illumina sequencing

Sample	A1-1	A1-2	A1-3	A2-1	A2-2	A2-3	A3-1	A3-2	A3-3	H1-1	H1-2	H1-3	H2-1	H2-2	H2-3	H3-1	H3-2	H3-3	All- Unigene
Total raw reads (Mb)	56.67	55.05	56.67	56.67	59.9	55.05	58.29	58.29	59.92	58.29	56.67	56.67	56.67	55.72	56.67	56.59	58.29	58.29	
Total clean reads (Mb)	45.08	44.62	45.23	44.57	44.22	44.53	45.35	45.22	44.09	45.27	45.38	45.22	44.78	43.69	44.48	44.82	44.42	45.36	
Q20 percentage (%)	98.09	98.19	98.12	98.07	98.12	98.18	98.25	98.1	98.78	98.09	98.17	98.21	98.13	98.08	98.1	98.18	98	98.22	
Transcripts																			
Total number	96236	82109	85747	63953	67018	71192	74902	74765	71737	71191	85909	78332	80222	62881	75889	77130	77221	81253	
Total length (bp)	64543033	58036174	60304688	45114838	48159734	50663988	47384945	53704506	44642571	48862194	56989258	49366623	50957902	40494895	52452952	53173862	51160551	53523302	
Mean length (bp)	670	706	703	705	718	711	632	718	622	686	663	630	635	643	691	689	662	658	
N50 (bp)	1151	1194	1198	1180	1220	1209	1012	1218	998	1118	1100	1029	1059	1040	1141	1128	1076	1069	
GC percentage (%)	41.45	41.54	41.75	41.56	41.41	41.56	41.96	41.9	42.26	41.7	41.67	41.98	41.88	42.05	42	41.87	41.95	41.82	
Unigene																			
Total number	64904	56882	59763	45453	47442	49670	53742	53282	51197	49574	58748	54935	55737	44682	53037	54392	54665	56800	155685
Total length (bp)	51754334	47244840	49182706	37389176	39897201	41600392	38828973	44364340	36388488	40388023	46299054	40225863	41441195	33446705	43281683	43899050	42176287	44044377	140639935
Mean length (bp)	797	830	822	822	840	837	722	832	710	814	788	732	743	748	816	807	771	775	903
N50 (bp)	1334	1349	1344	1324	1375	1373	1126	1344	1112	1272	1259	1175	1227	1175	1289	1262	1212	1209	1525
GC percentage (%)	41.51	41.6	41.78	41.64	41.51	41.6	41.98	41.92	42.29	41.77	41.72	42.03	41.94	42.09	42.03	41.89	41.98	41.86	40.72

**Table 3:** Statistics on the number of unigenes annotated with seven databases

Database	Number	Percentage
Nr-Annotated	68,773	44.17%
Nt-Annotated	63,118	40.54%
Swissprot-Annotated	45,356	29.13%
KEGG-Annotated	50,268	32.29%
COG-Annotated	25,540	16.40%
Interpro-Annotated	45,863	29.46%
GO-Annotated	12,237	7.86%
Overall	79,140	50.83%
Total	1,55,685	100%

**Table 4:** Histone proteins related genes involved in seed formation of Paeonia lutea

Stage	DEGs	Annotation	A-	H-	log_2_Fold Change
			Expression	Expression	(H/A)
A1 vs. H1	Unigene4684	histone H2B	120.9	9.3	-3.7
	Unigene18226	histone H2B	142.2	10	-3.8
	Unigene5759	histone H2B	88.4	5.9	-3.9
	Unigene5804	histone H2B	110.3	6.9	-4
	Unigene19081	histone H2B	57.4	3.5	-4.1
	Unigene14110	histone H2A	84.6	6.5	-3.7
	Unigene38241	histone H3	228.7	16.2	-3.8
	Unigene38235	histone H3	226.6	14.4	-4
	Unigene38223	histone H3	156	10.7	-3.9
	CL10135.Contig2	histone H1	40	2	-4.3
	CL10135.Contig5	histone H1	45.4	3	-3.9
A2 vs. H2	CL4680.Contig8	histone H4	6.6	96.6	3.9
	Unigene3742	histone H4	3	48.6	4
	Unigene12943	histone H2B	4.1	63.3	3.9
	Unigene16031	histone H2B	3.1	49.4	4
	Unigene18226	histone H2B	5.8	101.4	4.1
	Unigene38231	histone H3	5	77.8	4
	Unigene38223	histone H3	5.4	83.5	4
	Unigene38208	histone H3	3.3	53.2	4
A3 vs. H3	CL12701.Contig1	Histone deacetylase HDT1	1.8	38	4.4
	CL10318.Contig1	histone-binding protein RBBP4	3.7	105.3	4.8
	CL215.Contig1	histone H1	2.4	155.7	6

**Table 5:** Proline-rich extensin proteins related genes involved in seed formation of Paeonia lutea

Stage	DEGs	Annotation	A-	H-	log_2_Fold Change
			Expression	Expression	(H/A)
A1 vs. H1	CL4520.Contig2	extensin-like region protein	0.5	12.2	4.6
	Unigene9091	proline-rich extensin-like protein	1.6	35.6	4.5
	Unigene8849	proline-rich protein DC2	133.3	8.4	-4
	CL4042.Contig1	extensin-like (cell wall protein gp1)	42.4	0.7	-6
	CL2588.Contig3	proline-rich extensin-like protein	68.1	3.6	-4.2
	CL2588.Contig1	proline-rich protein	419.1	21.8	-4.3
	CL2588.Contig11	proline-rich extensin-like protein	128.1	6.4	-4.3
	CL2588.Contig2	proline-rich protein	333.7	21	-4
A2 vs. H2	CL2588.Contig2	proline-rich protein	6.2	155.4	4.6
	CL2588.Contig9	proline-rich protein	7.2	187	4.7
A3 vs. H3	CL15573.Contig4	extensin-3 like	412.1	37.7	-3.5
	CL15573.Contig10	extensin-3 like	90.1	13.6	-2.7
	CL15573.Contig1	extensin-3 like	780.5	126.4	-2.6
	Unigene12663	extensin-2-like	141.8	16.2	-3.1
	Unigene9091	proline-rich extensin-like protein	3705.8	447.5	-3
	Unigene9294	extensin-3-like	386.8	53.7	-2.8
	Unigene15381	extensin-3 like	345.3	50.6	-2.8
	Unigene13035	extensin like	131.7	20.2	-2.7
